# Supplementary material for: A conserved megaprotein-based molecular bridge critical for lipid trafficking and cold resilience
Source: Nat Commun. 2022 Nov 10;13:6805. doi: 10.1038/s41467-022-34450-y (PMC9649747; doi:10.1038/s41467-022-34450-y)
Supplement: Supplementary file 1 — Supplementary Information [file 41467_2022_34450_MOESM1_ESM.pdf]

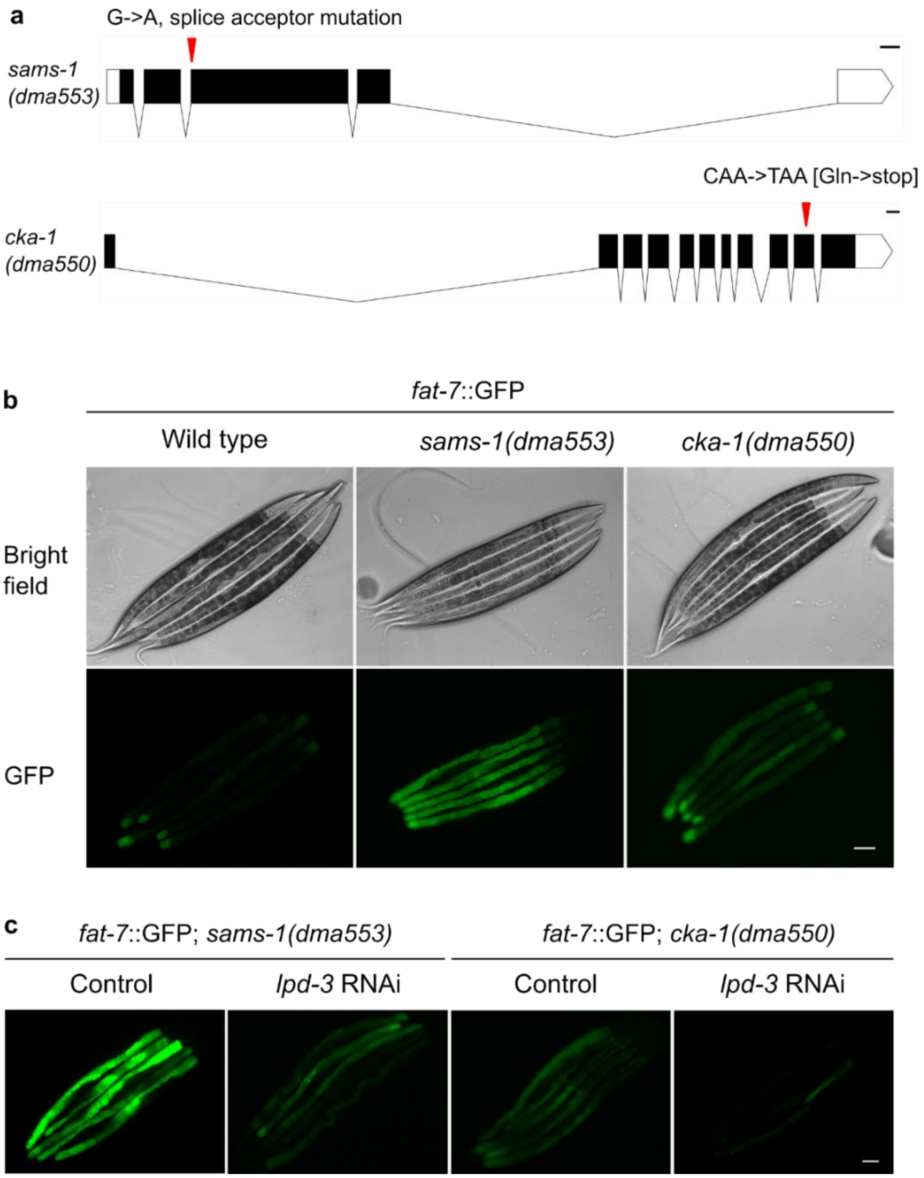

Supplementary Figure 1. Genetic identification of *cka-1* and *sams-1* in regulating *fat-7*::GFP. a, Gene diagrams showing *sams-1*(*dma553*) and *cka-1*(*dma550*) that cause a splice acceptor mutation and a premature stop codon mutation, respectively, identified by WGS. **b**, Representative bright-field and epifluorescence images showing activation of *fat-7*::GFP by *sams-1*(*dma553*) and *cka-1*(*dma550*). Both mutations were isolated from EMS screens and outcrossed at least 5 times, segregating with *fat-7*::GFP

activation phenotypes. **c**, Representative epifluorescence images showing that RNAi against *lpd-3* attenuated activation of *fat-7*::GFP by *sams-1*(*dma553*) or *cka-1*(*dma550*). Scale bar: 50 µm.

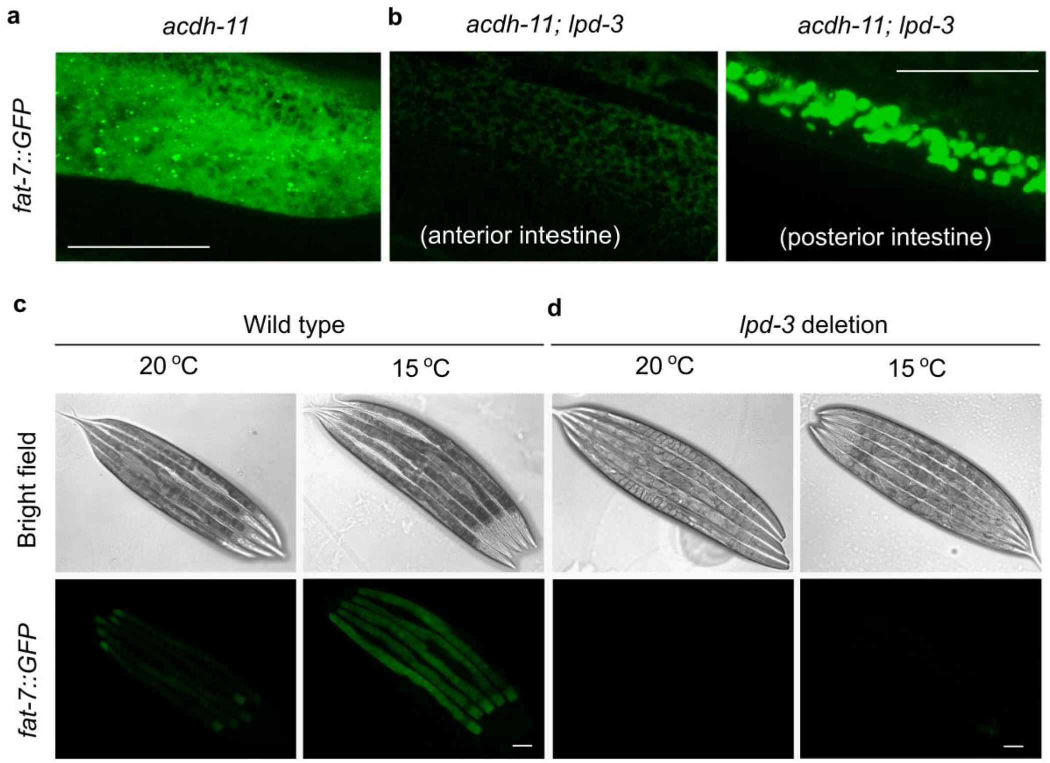

Supplementary Figure 2. *lpd-3* suppresses *acdh-11* or hypothermia-induced *fat-7*::GFP. a, Confocal fluorescence image (Z-stack) showing *fat-7*::GFP in *acdh-11(n5857)* mutants. **b**, Confocal fluorescence images showing *lpd-3(ok2138)* deletion mutation suppresses *fat-7*::GFP in *acdh-11(n5857)* mutants, more prominently in the anterior than posterior intestine. **c**, Bright field and epifluorescence images showing up-regulation of *fat-7*::GFP by hypothermia (15 °C 24 hrs). **d**, Bright field and epifluorescence images showing up-regulation of *fat-7*::GFP by hypothermia (15 °C 24 hrs) is blocked in *lpd-3(ok2138)* deletion mutants. Scale bar: 50 µm.

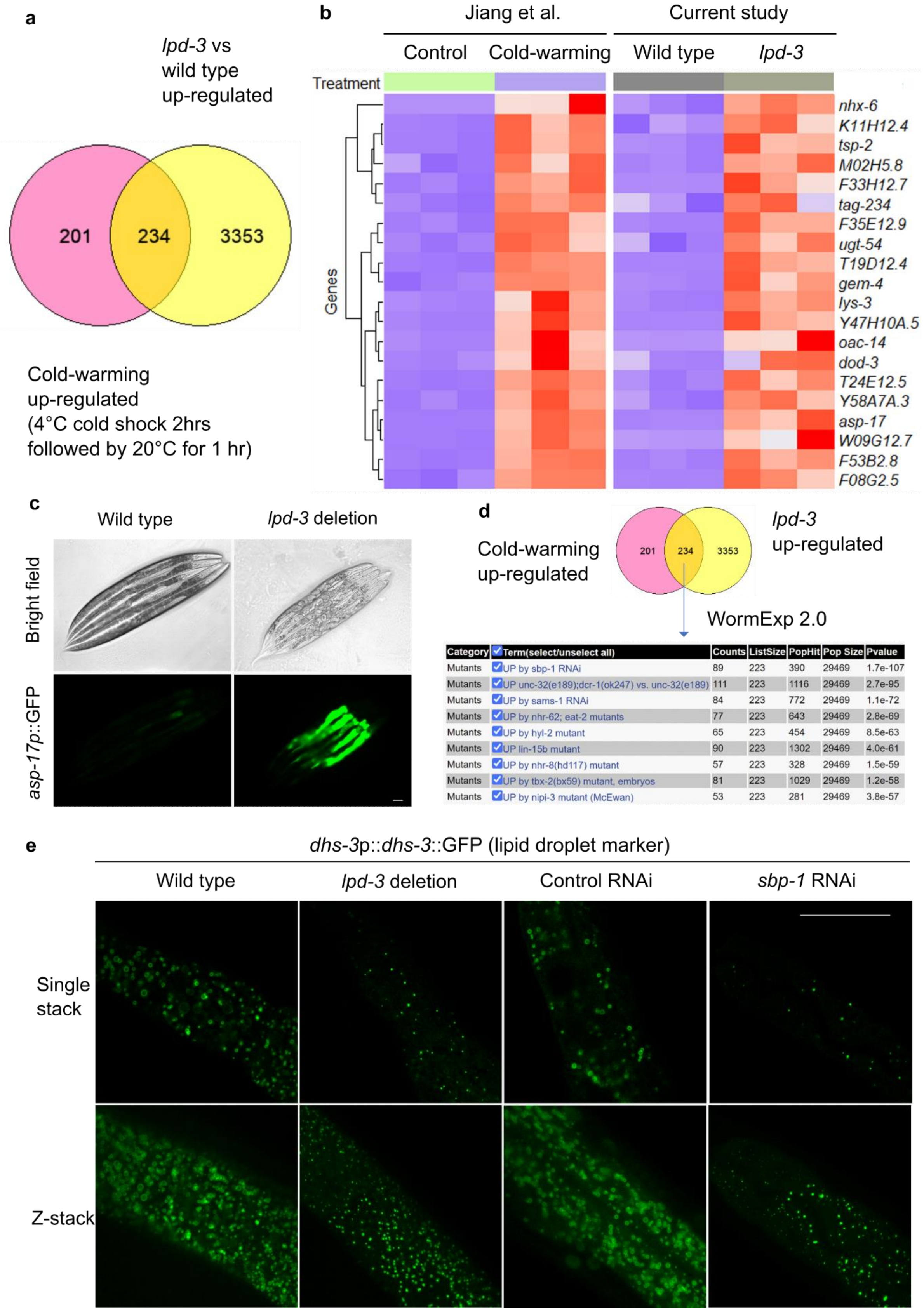

Category	Term(select/unselect all)	Counts	ListSize	PopHit	Pop Size	Pvalue
Mutants	UP by sbp-1 RNAi	89	223	390	29469	1.7e-107
Mutants	UP unc-32(e189);dcr-1(ok247) vs. unc-32(e189)	111	223	1116	29469	2.7e-95
Mutants	UP by sams-1 RNAi	84	223	772	29469	1.1e-72
Mutants	UP by nhr-62; eat-2 mutants	77	223	643	29469	2.8e-69
Mutants	UP by hyl-2 mutant	65	223	454	29469	8.5e-63
Mutants	UP lin-15b mutant	90	223	1302	29469	4.0e-61
Mutants	UP by nhr-8(hd117) mutant	57	223	328	29469	1.5e-59
Mutants	UP by tbx-2(bx59) mutant, embryos	81	223	1029	29469	1.2e-58
Mutants	UP by nipi-3 mutant (McEwan)	53	223	281	29469	3.8e-57

Supplementary Figure 3. RNAseq reveals genes commonly regulated by LPD-3, cold stress and SBP-1. a, Venn diagram showing commonly regulated genes by both *lpd-3(ok2138)* and cold stress. **b**, Heat map showing the top 30 regulated genes by *lpd-3(ok2138)* that are also regulated by cold stress. **c**, Bright field and epifluorescence images showing constitutive up-regulation of *asp-17p::*GFP in *lpd-3(ok2138)* mutants. Scale bar: 50 µm. **d**, WormExp (https://wormexp.zoologie.uni-kiel.de/wormexp/) analysis of *lpd-3* and cold-commonly regulated genes showing they are most similar to the gene set regulated by RNAi against *sbp-1*. *P* values were calculated by the program EASE based on the Fisher Exact test by the WormExp webpage. **e**, Representative confocal fluorescence images (Single stack, above; Z-stack, below) showing that both *lpd-3(ok2138)* and *sbp-1* RNAi can cause fewer in numbers and smaller in size of lipid droplet makers *dhs-3*p::*dhs-3*::GFP. Scale bar: 50 µm.

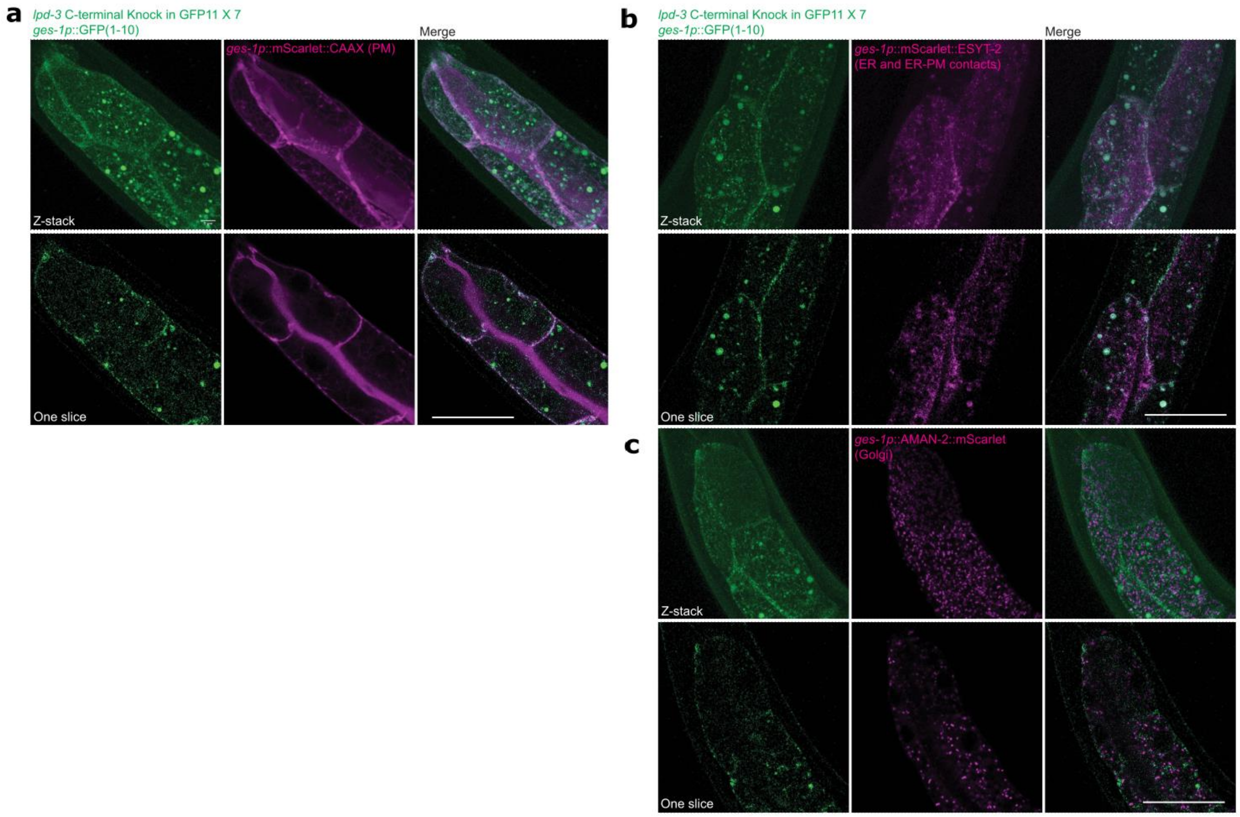

Supplementary Figure 4. Endogenous LPD-3 localizes to ER-PM membrane contact sites. a, Representative confocal fluorescence images showing endogenous LPD-3::GFP (generated by CRISPR/Cas9-mediated knock-in of 7xGFP11 and 7xGFP1-10 complementation) in apposition with mScarlet::CAAX(PM). **b**, Representative confocal fluorescence images showing endogenous LPD-3::GFP (generated by CRISPR/Cas9-mediated knock-in of GFP11 and 7xGFP1-10 complementation) in apposition with mScarlet::ESYT-2, a marker for ER-PM junctions. **c**, Representative confocal fluorescence images showing endogenous LPD-3::GFP (generated by CRISPR/Cas9-mediated knock-in of GFP11 and 7xGFP1-10 complementation) in apposition with AMAN-2::mScarlet, a Golgi marker. Scale bar, 50 µm.

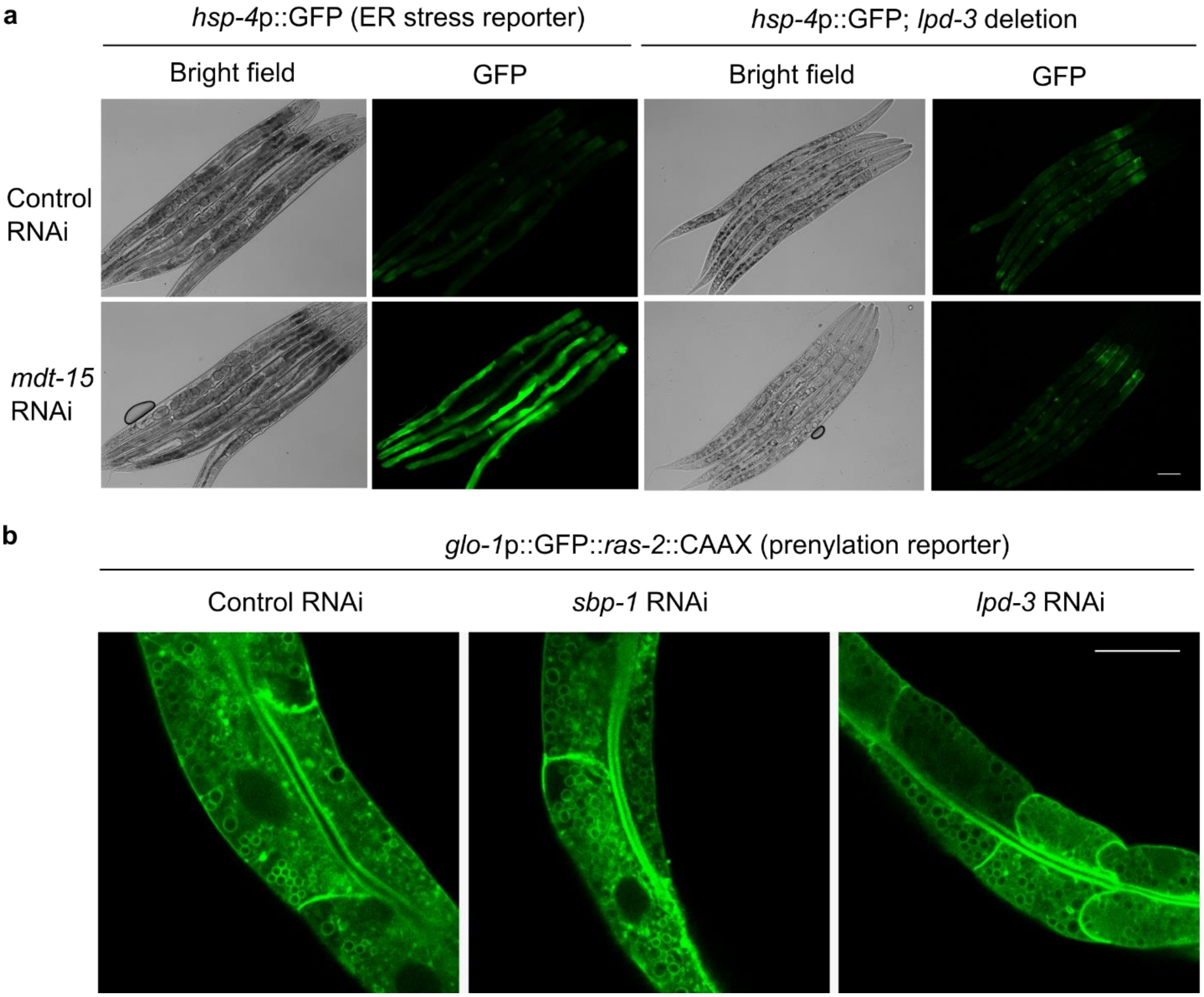

Supplementary Figure 5. Additional phenotypes of *lpd-3* mutants. a, Representative epifluorescence images showing activation of the *hsp-4*p::GFP ER stress reporter by RNAi against *mdt-15* in wild type but not *lpd-3(ok2138)* mutants. *mdt-15* RNAi activates *hsp-4*p::GFP because of reduced desaturase gene expression and excessive acyl chain saturation of ER membrane lipids[47]. **b,** Representative confocal fluorescence images showing largely normal intestinal PM morphology and PM-targeted trafficking of the prenylation reporter *glo-1*p::GFP::*ras-2*::CAAX in *sbp-1* or *lpd-3* RNAi-treated animals. Scale bar: 50 µm.

a

Compounds	Rescue of *lpd-3* mutants
M9	-
M9 + Lecithin	++++
M9 + soy phospholipids	+++++
M9 + egg phospholipids	+++++
M9 + phosphotidylcholine	+++
M9 + choline	-
M9 + ethanolamine	-
M9 + serine	-
M9 + oleic acid salts	+
M9 + stearic acid salts	-

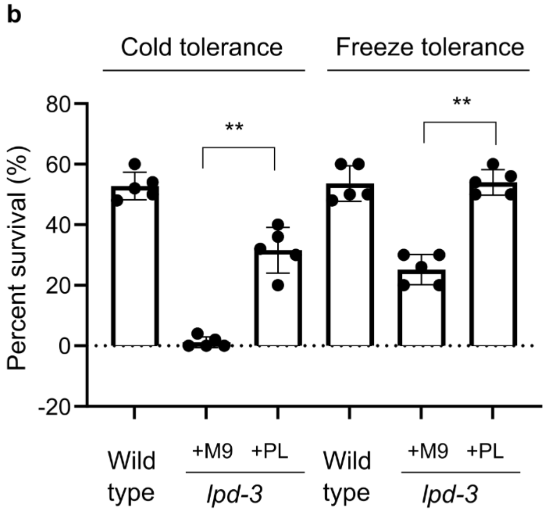

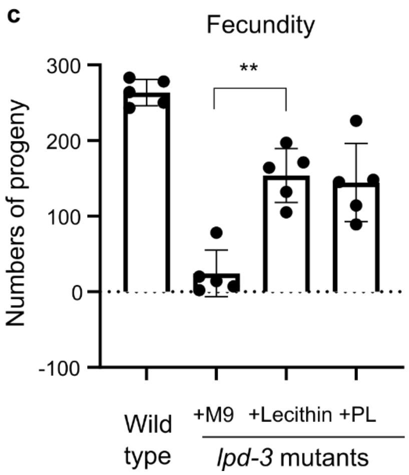

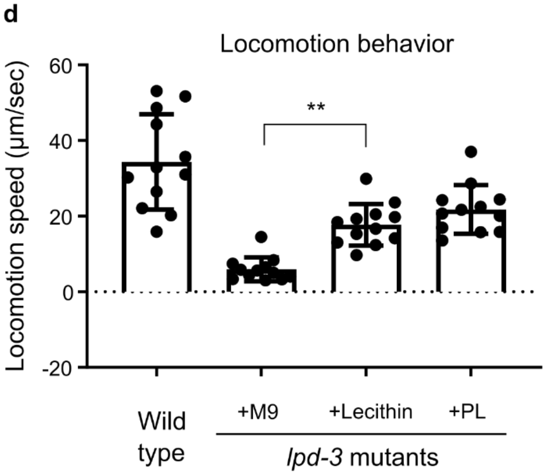

Supplementary Figure 6. Phospholipid/Lecithin rescue of various *lpd-3* mutant phenotypes. a, Table summary for the degrees of rescue (indicated by the numbers of + sign) of developmental delay in *lpd-3* mutants by Lecithin, phospholipids derived from soy or egg, or various other phospholipid constituent compounds. **b**, Quantification of percent survival of wild type or *lpd-3* mutants, and rescue by soy phospholipid (PL), in the cold or freezing tolerance assays. Values are means ± S.D with $**P < 0.01$ (N = 5 independent experiments, one-way ANOVA followed by multiple two-sided unpaired t-tests). **c**, Quantification of fecundity (numbers of progeny per hermaphrodite) of wild type or *lpd-3* mutants, and rescue by soy phospholipid (PL) or Lecithin. Values are means ± S.D with $**P < 0.01$ (N = 5 independent experiments, one-way ANOVA followed by multiple two-sided unpaired t-tests). **d**, Quantification of locomotion behavior (average speed of young adult hermaphrodite) of wild type or *lpd-3* mutants, and

rescue by soy phospholipid (PL) or Lecithin. Values are means ± S.D with **$P < 0.01$ (N = 12 animals per group, one-way ANOVA followed by multiple two-sided unpaired t-tests). Source data are provided as a Source Data file.

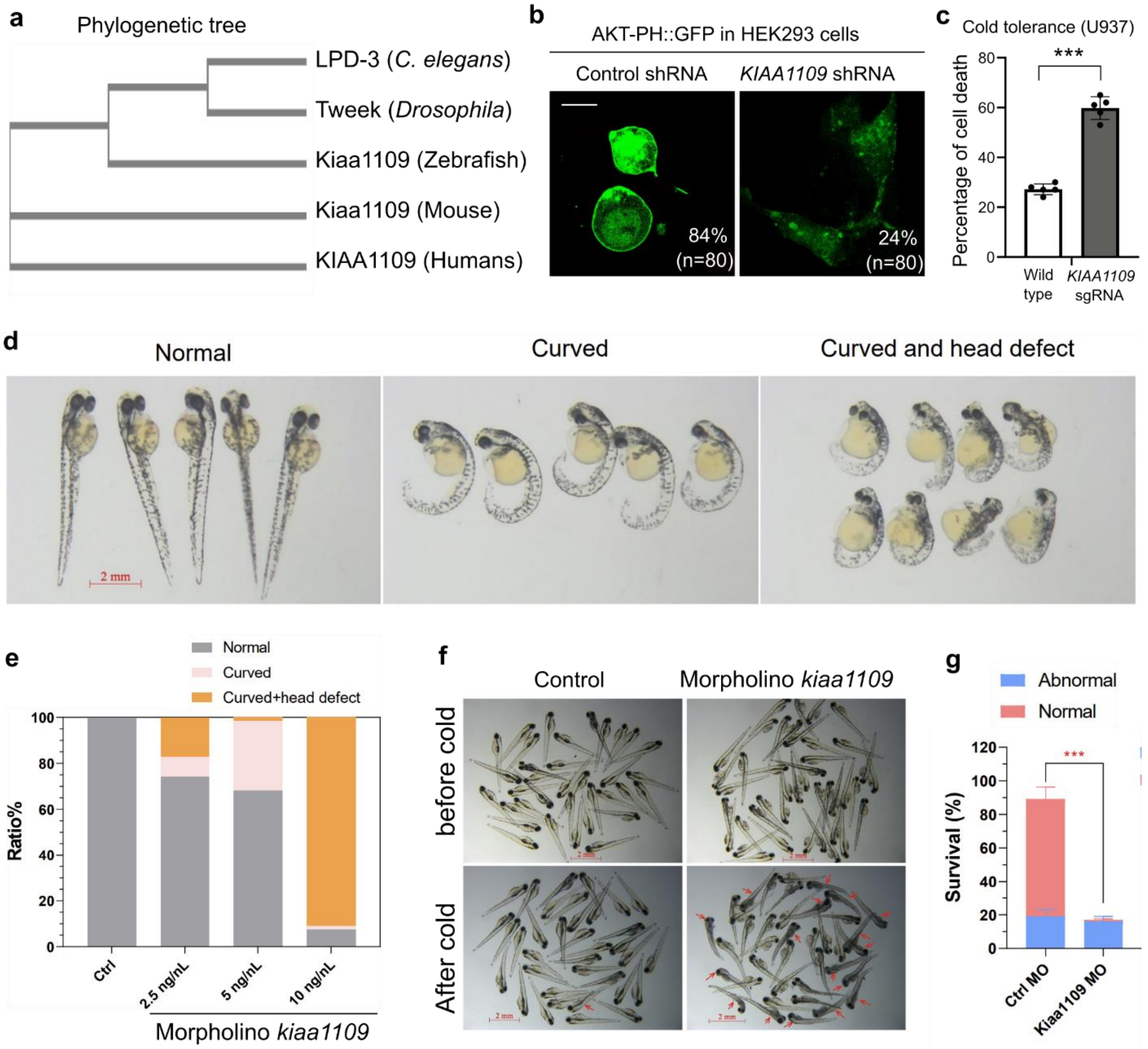

Supplementary Figure 7. Evidence for conserved roles of LPD-3 protein families in lipid trafficking and cold tolerance. a, Clustal Omega-generated phylogenetic tree of the LPD-3 protein family from major metazoan species (*C. elegans*, *Drosophila*, Zebrafish, mouse and humans). **b**, Representative

confocal fluorescence imaging of AKT-PH::GFP (PIP3 binding) in HEK293 cells co-transfected with the AKT-PH::GFP and shRNA (control or *KIAA1109* with 73% knockdown efficiency, SIGMA) plasmids, showing reduced plasma membrane localization of AKT-PH::GFP by shRNA against *KIAA1109.* Percentages of cells with normal membrane-localized fluorescent signals were noted for shRNA control and sh-*KIAA1109.* Scale bar: 10 µm. **c**, Percentage of cell death after cold shock (4 ºC for 20 hrs) in wild type and *KIAA1109* KO cells generated by CRISPR/Cas9. Values are means ± S.D with ***, $P < 0.001$; N = 5 independent experiments (n > 400 cells analyzed by SYTOX Green for each experiment). **d**, Representative images of zebrafish embryos showing various morphological phenotypes caused by morpholino against *kiaa1109.* **e**, Quantification of the various morphological phenotypes caused by morpholino against *kiaa1109.* **f**, Representative images of zebrafish embryos showing a phenotype of reduced cell survival caused by morpholino against *kiaa1109.* **g**, Quantification of reduced cell survival caused by morpholino against *kiaa1109.* Values are means ± S.D with ***, $P < 0.001$; N = 6 independent experiments (n > 30 fish for each experiment). Source data are provided as a Source Data file.
