# Supplementary material for: A conserved megaprotein-based molecular bridge critical for lipid trafficking and cold resilience
Source: Nat Commun. 2022 Nov 10;13:6805. doi: 10.1038/s41467-022-34450-y (PMC9649747; doi:10.1038/s41467-022-34450-y)
Supplement: Supplementary file 3 — Description of Additional Supplementary Files [file 41467_2022_34450_MOESM3_ESM.pdf]

File name: Supplementary Data 1

Description: RNAseq identified differentially expressed genes in *lpd-3* mutants.

File name: Supplementary Data 2

Description: Protein Database Bank (pdb) file for the predicted LPD-3 structure.
