# Supplementary material for: A conserved megaprotein-based molecular bridge critical for lipid trafficking and cold resilience
Source: Nat Commun. 2022 Nov 10;13:6805. doi: 10.1038/s41467-022-34450-y (PMC9649747; doi:10.1038/s41467-022-34450-y)
Supplement: Supplementary file 6 — Reporting Summary [file 41467_2022_34450_MOESM6_ESM.pdf]

nature portfolio

Corresponding author(s): Dengke K. Ma

Last updated by author(s): 10/15/2022

Reporting Summary

Nature Portfolio wishes to improve the reproducibility of the work that we publish. This form provides structure for consistency and transparency in reporting. For further information on Nature Portfolio policies, see our Editorial Policies and the Editorial Policy Checklist.

Statistics

For all statistical analyses, confirm that the following items are present in the figure legend, table legend, main text, or Methods section.

n/a	Confirmed	
☐	☒	The exact sample size (*n*) for each experimental group/condition, given as a discrete number and unit of measurement
☐	☒	A statement on whether measurements were taken from distinct samples or whether the same sample was measured repeatedly
☐	☒	The statistical test(s) used AND whether they are one- or two-sided<br>*Only common tests should be described solely by name; describe more complex techniques in the Methods section.*
☒	☐	A description of all covariates tested
☒	☐	A description of any assumptions or corrections, such as tests of normality and adjustment for multiple comparisons
☐	☒	A full description of the statistical parameters including central tendency (e.g. means) or other basic estimates (e.g. regression coefficient) AND variation (e.g. standard deviation) or associated estimates of uncertainty (e.g. confidence intervals)
☐	☒	For null hypothesis testing, the test statistic (e.g. *F*, *t*, *r*) with confidence intervals, effect sizes, degrees of freedom and *P* value noted<br>*Give P values as exact values whenever suitable.*
☒	☐	For Bayesian analysis, information on the choice of priors and Markov chain Monte Carlo settings
☒	☐	For hierarchical and complex designs, identification of the appropriate level for tests and full reporting of outcomes
☒	☐	Estimates of effect sizes (e.g. Cohen's *d*, Pearson's *r*), indicating how they were calculated

Our web collection on statistics for biologists contains articles on many of the points above.

Software and code

Policy information about availability of computer code

Data collection	Fluorescence and bright-field imaging data were collected by the LAS X Life Science Microscope Software and ZEN lite image processing software. RNAseq data were collected by the DNBSEQ sequencing platform at BGI with 10-20M paired end reads per sample.
Data analysis	RNAseq data were analyzed by the DESeq2 package. Statistic analysis and plots of data were generated with GraphPad Prism 9. Structure prediction of each fragment of LPD-3 was generated by AlphaFold v2.0 (https://cryonet.ai/af2/) program. Predicted structures were aligned using Chimera based on the overlapping sequence. The aligned structures of all fragments were merged in Coot to obtain the full-length structure. C-terminal flexible loop was manually adjusted. The structural images were prepared in ChimeraX.

For manuscripts utilizing custom algorithms or software that are central to the research but not yet described in published literature, software must be made available to editors and reviewers. We strongly encourage code deposition in a community repository (e.g. GitHub). See the Nature Portfolio guidelines for submitting code & software for further information.

Data

Policy information about availability of data

All manuscripts must include a data availability statement. This statement should provide the following information, where applicable:

- Accession codes, unique identifiers, or web links for publicly available datasets
- A description of any restrictions on data availability
- For clinical datasets or third party data, please ensure that the statement adheres to our policy

The RNAseq read datasets were deposited in NCBI SRA (Sequence Read Archive) under the BioProject accession PRJNA827259. All other data generated for this study are included in this article, including those used for WormExp 2.0 (https://wormexp.zoologie.uni-kiel.de/wormexp/) and AlphaFold v2.0 (https://cryonet.ai/af2/).

Field-specific reporting

Please select the one below that is the best fit for your research. If you are not sure, read the appropriate sections before making your selection.

☒ Life sciences ☐ Behavioural & social sciences ☐ Ecological, evolutionary & environmental sciences

For a reference copy of the document with all sections, see nature.com/documents/nr-reporting-summary-flat.pdf

Life sciences study design

All studies must disclose on these points even when the disclosure is negative.

Sample size	Sample sizes were chosen based on our previous experiences in the C. elegans assays and aided with a power analysis for probability of finding statistically significant results for given population effect sizes.
Data exclusions	No data were excluded for analysis.
Replication	Each figure contains the number of animals used or biological replicates used.
Randomization	Across experiments, C. elegans animals of given genotype were randomly selected for analysis. Worms grouped per genotype were cultivated by the standard method and placed randomly on growth plates under specified conditions. To synchronize worms, L4-stage worms were randomly picked under the stereo-microscope onto new plates. Adult worms of specified ages were then randomly collected and analyzed by imaging, survival and gene expression assays and analyses. See details in Method.
Blinding	For imaging and phenotypic analyses, the experiments were not blinded since the mutant phenotype (pale etc.) was observed in an objective manner under a microscope followed by genotyping confirmation by PCR and Sanger sequencing to ensure the correct experimental setup. Processing of the samples for RNAseq was performed by independent researchers in the BGI sequencing facilities in a blinded manner. All measurements and analyses were conducted by multiple repeats and investigators to confirm the observed phenotypes.

Reporting for specific materials, systems and methods

We require information from authors about some types of materials, experimental systems and methods used in many studies. Here, indicate whether each material, system or method listed is relevant to your study. If you are not sure if a list item applies to your research, read the appropriate section before selecting a response.

Materials & experimental systems

n/a	Involved in the study	
☒	☐	Antibodies
☐	☒	Eukaryotic cell lines
☒	☐	Palaeontology and archaeology
☐	☒	Animals and other organisms
☒	☐	Human research participants
☒	☐	Clinical data
☒	☐	Dual use research of concern

Methods

n/a	Involved in the study	
☒	☐	ChIP-seq
☒	☐	Flow cytometry
☒	☐	MRI-based neuroimaging

Eukaryotic cell lines

Policy information about cell lines

Cell line source(s)	ATCC
Authentication	Cell lines were received from ATCC authenticated with short tandem repeat (STR) profiling.
Mycoplasma contamination	Cell lines were not tested for Mycoplasma contamination as no indications of contamination were observed.
Commonly misidentified lines (See ICLAC register)	N/A

Animals and other organisms

Policy information about studies involving animals; ARRIVE guidelines recommended for reporting animal research

Laboratory animals	An invertebrate animal C. elegans was used throughout the study and grown under standard laboratory conditions on NGM plates at 20°C unless otherwise specified. MEFs were derived from Kiaa1109 mutant mice [B6N(Cg)-4932438A13Riktm1b(EUCOMM)Hmgu/J, Stock No.026878] generated by the Knockout Mouse Project (KOMP) at The Jackson Laboratory (Bar Harbor, Maine, USA) in a facility

	with 12 light/12 dark cycle, 65-75°F and 40-60% humidity. All experimental protocols followed National Institutes of Health (NIH) guidelines and were approved by the University of Texas Southwestern Institutional Animal Care and Use Committee.
Wild animals	N/A
Field-collected samples	N/A
Ethics oversight	N/A

Note that full information on the approval of the study protocol must also be provided in the manuscript.
